# An Algorithm of *l*_1_-Norm and *l*_0_-Norm Regularization Algorithm for CT Image Reconstruction from Limited Projection

**DOI:** 10.1155/2020/8873865

**Published:** 2020-08-28

**Authors:** Xiezhang Li, Guocan Feng, Jiehua Zhu

**Affiliations:** ^1^Department of Mathematical Sciences, Georgia Southern University, Statesboro 30460, USA; ^2^School of Mathematics, Sun Yat-sen University, Guangzhou 510275, China

## Abstract

The *l*_1_-norm regularization has attracted attention for image reconstruction in computed tomography. The *l*_0_-norm of the gradients of an image provides a measure of the sparsity of gradients of the image. In this paper, we present a new combined *l*_1_-norm and *l*_0_-norm regularization model for image reconstruction from limited projection data in computed tomography. We also propose an algorithm in the algebraic framework to solve the optimization effectively using the nonmonotone alternating direction algorithm with hard thresholding method. Numerical experiments indicate that this new algorithm makes much improvement by involving *l*_0_-norm regularization.

## 1. Introduction

Computed tomography (CT) has been widely applied in medical imaging and industry for over decades. In some applications, the reconstructions do not require a high-quality image, or complete projection data for exact reconstructions is not available because of the scanning conditions or radiation issue. CT reconstruction from limited projection data is of particular importance, and statistical and iterative reconstruction algorithms outperform the analytic approaches in these applications.

The theory of compressed sensing [1, 2] has provided a novel framework for CT image reconstruction from limited projection data with the prior knowledge of the sparse gradients for CT images. The total variation (TV) based regularization [3] for CT image reconstruction is connected to compressed sensing under the sparse transform of the gradient operator [4, 5]. The TV of an image is the *l*_1_-norm of the gradients of the image, so the TV minimization is also known as the *l*_1_-minimization.

The *l*_1_-norm-based regularization can greatly reduce the streak artifacts arising from few-view CT [6]. However, the results are over smoothing and lose some detailed features including contrast that causes edge blurred [7]. In addition, the *l*_1_-norm regularization does not provide a representation sparse enough. To overcome the drawback, many improved TV-based reconstruction algorithms have been developed. One of them is the reweighted *l*_1_ minimization [8]. Algorithms for regularization involving multiple norms are also developed. For example, the alternating direction method (ADM) [9, 10] and the nonmonotone alternating direction algorithm (NADA) [11, 12] are proposed for solving an *l*_1_-norm minimization with an *l*_2_-norm constraint.

The *l*_0_-norm of an image vector, defined as the number of its nonzero elements, measures the sparsity of a vector appropriately. So the *l*_0_-norm-based regularization for CT represents the sparsity of the gradient image better than the *l*_1_-norm-based regularization and preserves the edge while suppressing the streak artifacts [13]. However, the *l*_0_-norm is a nonconvex function and the *l*_0_-norm regularization problem is NP hard [14]. So many variants of the regularization algorithms are developed. An edge-preserving image reconstruction method for limited-angle CT is investigated based on *l*_0_-norm regularized gradient prior [15]. An imaging reconstruction model for few-view CT based on *l*_0_ sparse regularization is proposed [16]. A new *l*_0_ regularization with wavelet tight framelets is addressed to suppress the slope artifacts in the limited-angle X-ray CT reconstruction [17]. Regularization involving multiple norms is also developed to address the individual drawbacks of the *l*_1_-norm and *l*_0_-norm. An image reconstruction model based on *l*_0_-norm and *l*_2_-norm regularization for the limited-angle CT is proposed [18]. Numerical experiments indicate that the algorithm has the advantage in suppressing slope artifacts. A combined smoothed *l*_0_-norm and *l*_1_-norm regularization algorithm using the NADA method for CT image reconstruction is proposed and demonstrated to have better performance than the *l*_1_-norm regularization [19].

In this paper, we will develop a new combined *l*_1_-norm and *l*_0_-norm regularization model for CT image reconstruction from limited projection data. The main contributions of this paper are as follows:

1. We generalize the *l*_1_-norm regularization to an *l*_1_-norm and *l*_0_-norm regularization for CT image reconstruction from limited projection data. The proposed model can achieve superior performance compared with the existing related methods for multiple reasons. Firstly, due to the *l*_1_-norm regularization term, the proposed model can reduce streak artifacts in limited-data CT. Secondly, due to the *l*_0_-norm regularization term, the oversmoothing from the *l*_1_-norm regularization term is improved. The proposed model is less smoothing, thus better edge-preserving, and provides a better sparsity representation. Thirdly, since the *l*_0_-norm is adopted without using the smoothed *l*_0_-norm approximation, the proposed model has the advantage in suppressing slope artifacts
2. We propose an algorithm in the algebraic framework to solve the optimization effectively using the nonmonotone alternating direction algorithm with hard thresholding (NADA-HT) method

The rest of the paper is organized as follows. In Section 2, we present a combined *l*_1_-norm and *l*_0_-norm-based regularization model and propose an algorithm for CT image reconstruction from limited projection data. Numerical experiments for a comparison of regularization models with/without the *l*_0_-norm and the discussion are given in Section 3.

## 2. Materials and Methods

The projection data in CT can be modelled as a linear system,

(1) $$\begin{array}{c} \Phi f+e=u, \end{array}$$

where *Φ* is an *m* × *n*^2^ projection matrix, *f* ∈ *R*^*n*^2^^ represents a 2D *n* × *n* image to be reconstructed, *e* an additive noise with ‖*e*‖_2_ ≤ *ε* for some known *ε* > 0, and *u* ∈ *R*^*m*^ the noisy projection data. For limited data reconstruction, the underdetermined system (*m* ≪ *n*^2^) has infinitely many solutions. An optimal solution representing the original image as good as possible can be sought by minimizing the energy function with a TV regularization term [8]:

(2) $$\begin{array}{c} \underset{f}{\min\;}\;{\left\|f\right\|}_{TV}\text{ subject to }\Phi f+e=u, \end{array}$$

where the total variation ‖*f*‖_*TV*_ is the *l*_1_-norm of the magnitude of the discrete gradients,

(3) $$\begin{array}{c} {\left\|f\right\|}_{TV}={\left\|\nabla f\right\|}_{1}=\underset{i,j}{\sum }\sqrt{{\left(f_{i+1,j}-f_{i,j}\right)}^{2}+{\left(f_{i,j+1}-f_{i,j}\right)}^{2}}. \end{array}$$

Then, a 2D image *f* with sparse gradients and noisy measurements in CT can be reconstructed by solving the following *l*_1_-norm minimization with an *l*_2_-norm constraint,

(4) $$\begin{array}{c} \underset{f}{\min\;}{\left\|\nabla f\right\|}_{1}\text{ subject to}{\left\|\Phi f-u\right\|}_{2}^{2}\leq \varepsilon . \end{array}$$

However, the image reconstructed by solving (4) has slope artifacts near the edge of the object for limited-angle projection data [20].

We consider the following minimization model

(5) $$\begin{array}{c} \underset{f}{\min\;}\left\{{\left\|\nabla f\right\|}_{1}+\alpha {\left\|\nabla f\right\|}_{0}+\frac{\gamma }{2}{\left\|f\right\|}_{2}^{2}\right\}\text{ subject to}{\left\|\Phi f-u\right\|}_{2}^{2}\leq \varepsilon , \end{array}$$

where ‖∇*f*‖_0_ stands for the number of nonzero gradients (∇*f*)_*p*_, 1 ≤ *p* ≤ *n*^2^. The term (*γ*/2)‖*f*‖_2_^2^, *γ*⟶0+, makes the energy of reconstructed image to be minimized and reduces the ill-posedness of CT reconstruction [18].

For convenience, we denote *D*_*p*_*f* as the forward difference of *f* at a pixel *p* in both horizontal and vertical directions, i.e.,

(6) $$\begin{array}{c} D_{p}f=D_{i,j}f={\left[f_{i+1,j}-f_{i,j},f_{i,j+1}-f_{i,j}\right]}^{T}\in R^{2},\text{for }p=\left(i-1\right)n+j. \end{array}$$

Then, (∇*f*)_*p*_ = *D*_*p*_*f* for 1 ≤ *p* ≤ *n*^2^ and ‖∇*f*‖_1_ = ∑_*p*=1_^*n*^2^^‖*D*_*p*_*f*‖_2_.

Minimization (5) is equivalent to

(7) $$\begin{array}{c} \underset{f}{\min\;}\left\{\frac{\beta }{2}{\left\|\Phi f-u\right\|}_{2}^{2}+\overset{n^{2}}{\underset{p=1}{\sum }}\left({\left\|D_{p}f\right\|}_{2}+\alpha {\left\|D_{p}f\right\|}_{0}\right)+\frac{\gamma }{2}{\left\|f\right\|}_{2}^{2}\right\}, \end{array}$$

where the notation ‖*D*_*p*_*f*‖_0_ is set to be 1 for nonzero *D*_*p*_*f*, otherwise 0. The above minimization can be converted, with a parameter *β* > 0 and vectors *v*_*p*_′s, to

(8) $$\begin{array}{c} \begin{array}{c} \underset{f}{\min\;}\left\{\frac{\beta }{2}{\left\|\Phi f-u\right\|}_{2}^{2}+\overset{n^{2}}{\underset{p=1}{\sum }}\left({\left\|v_{p}\right\|}_{2}+\alpha {\left\|v_{p}\right\|}_{0}\right)+\frac{\gamma }{2}{\left\|f\right\|}_{2}^{2}\right\}\\ \text{subject to }D_{p}f=v_{p},\;1\leq p\leq n^{2}. \end{array} \end{array}$$

Adapting Lagrangian vectors *λ*_*p*_ ∈ *R*^2^, 1 ≤ *p* ≤ *n*^2^, we convert Minimization (8) into the following problem using the augmented Lagrangian method

(9) $$\begin{array}{c} \underset{f}{\min\;}\left\{\frac{\beta }{2}{\left\|\Phi f-u\right\|}_{2}^{2}+\overset{n^{2}}{\underset{p=1}{\sum }}\left({\left\|v_{p}\right\|}_{2}+\alpha {\left\|v_{p}\right\|}_{0}+\frac{\mu }{2}{\left\|D_{p}f-v_{p}+\lambda_{p}\right\|}_{2}^{2}\right)+\frac{\gamma }{2}{\left\|f\right\|}_{2}^{2}\right\}. \end{array}$$

For simplicity, we write *Df*,  *v*, and *λ* as vectors whose *p*-th components are *D*_*p*_*f*,  *v*_*p*_, and *λ*_*p*_, 1 ≤ *p* ≤ *n*^2^, respectively. Then, ‖*Df* − *v* + *λ*‖_2_^2^ = ∑_*p*=1_^*n*^2^^‖*D*_*p*_*f* − *v*_*p*_ + *λ*_*p*_‖_2_^2^. Thus, Minimization (9) becomes

(10) $$\begin{array}{c} \underset{f}{\min\;}\left\{\frac{\beta }{2}{\left\|\Phi f-u\right\|}_{2}^{2}+\overset{n^{2}}{\underset{p=1}{\sum }}\left({\left\|v_{p}\right\|}_{2}+\alpha {\left\|v_{p}\right\|}_{0}\right)+\frac{\mu }{2}{\left\|Df-v+\lambda \right\|}_{2}^{2}+\frac{\gamma }{2}{\left\|f\right\|}_{2}^{2}\right\}. \end{array}$$

Next, we will develop an algorithm for solving Minimization (10). By convention, Minimization (10) can be solved by ADM iteratively. The *k*-th step of the ADM involves three procedures,

(11) $$\begin{array}{c} f^{k+1}\in \arg\underset{f}{\min}\left\{\frac{\beta }{2}{\left\|\Phi f-u\right\|}_{2}^{2}+\frac{\mu }{2}{\left\|Df-v^{k}+\lambda^{k}\right\|}_{2}^{2}+\frac{\gamma }{2}{\left\|f\right\|}_{2}^{2}\right\}, \end{array}$$

(12) $$\begin{array}{c} v^{k+1}\in \arg\underset{v}{\min}\left\{\overset{n^{2}}{\underset{p=1}{\sum }}\left({\left\|v_{p}\right\|}_{2}+\alpha {\left\|v_{p}\right\|}_{0}\right)+\frac{\mu }{2}{\left\|{Df}^{k+1}-v+\lambda^{k}\right\|}_{2}^{2}\right\}, \end{array}$$

(13) $$\begin{array}{c} \lambda^{k+1}=\lambda^{k}-\left(v^{k+1}-{Df}^{k+1}\right). \end{array}$$

First, Minimization (11) can be solved by ADM with the gradient decent method involving nonmonotone line search, for example, the NADA.

Next, we seek for a solution of (12). Minimization (12) is equivalent to finding

(14) $$\begin{array}{c} v_{p}^{k+1}\in \arg\underset{v_{p}}{\min}\left\{{\left\|v_{p}\right\|}_{2}+\alpha {\left\|v_{p}\right\|}_{0}+\frac{\mu }{2}{\left\|D_{p}f^{k+1}-v_{p}+\lambda_{p}^{k}\right\|}_{2}^{2}\right\},\text{ for }1\leq p\leq n^{2}. \end{array}$$

We introduce a hard thresholds (HT) operator *H*_*κ*_(*w*) on *R*^2^ with the threshold *κ* defined as

(15) $$\begin{array}{c} H_{\kappa }\left(w\right)=\left\{\begin{array}{cc} 0 & \text{if }{\left\|w\right\|}_{2}\leq \kappa \\ w & \text{otherwise}. \end{array}\right. \end{array}$$

A solution *v*_*p*_^*k*+1^ is provided in the following theorem.

**Theorem 1 .**

Let *w* = *D*_*p*_*f*^*k*+1^ + *λ*_*p*_^*k*^ and $\kappa =\left(1/\mu \right)\left(1+\sqrt{2\mu \alpha }\right)$. If 2*μα* > 1, then a minimizer *v*_*p*_^*k*+1^ for Minimization (14) is given by

(16) $$\begin{array}{c} v_{p}^{k+1}=\max\left\{1-\frac{1}{\mu {\left\|w\right\|}_{2}},0\right\}H_{\kappa }\left(w\right). \end{array}$$

**Proof**

For simplicity, denote *z* = *v*_*p*_. The objective function in Minimization (14) is rewritten as

(17) $$\begin{array}{c} g\left(z\right)={\left\|z\right\|}_{2}+\alpha {\left\|z\right\|}_{0}+\frac{\mu }{2}{\left\|z-w\right\|}_{2}^{2}. \end{array}$$

Without loss of generality, we assume that *w* ≠ 0 since *z*_*opt*_ = 0 in the case of *w* = 0.

For *z* = 0, *g*(*z*) = (*μ*/2)‖*w*‖_2_^2^.

For *z* ≠ 0, *g*(*z*) = ‖*z*‖_2_ + *α* + (*μ*/2)‖*z* − *w*‖_2_^2^. The minimizer is derived in the following two cases.

- Case 1. *μ*||*w*||_2_ > 1

We claim that *g*_min_ = ‖*w*‖_2_ + *α* − 1/(2*μ*) at *z* = (1 − 1/(*μ*‖*w*‖_2_))*w*. In fact, it follows from

(18) $$\begin{array}{c} \frac{dg}{dz}=\frac{1}{{\left\|z\right\|}_{2}}z+\mu \left(z-w\right)=0 \end{array}$$

that a minimizer *z* is a scalar multiple of *w*. Let *z* = *tw* where *t* > 0 due to the minimization problem. Then, the function

(19) $$\begin{array}{c} h\left(t\right)≔g\left(z\right)=\frac{\mu }{2}{\left\|tw-w\right\|}_{2}^{2}+{\left\|tw\right\|}_{2}+\alpha =\frac{\mu }{2}{\left\|w\right\|}_{2}^{2}t^{2}-\left(\mu {\left\|w\right\|}_{2}^{2}-{\left\|w\right\|}_{2}\right)t+\frac{\mu }{2}{\left\|w\right\|}_{2}^{2}+\alpha \end{array}$$

reaches its minimum value

(20) $$\begin{array}{c} h_{\min}=\alpha +\frac{\mu }{2}{\left\|w\right\|}_{2}^{2}-\frac{{\left(\mu {\left\|w\right\|}_{2}^{2}-{\left\|w\right\|}_{2}\right)}^{2}}{2\mu {\left\|w\right\|}_{2}^{2}}=\alpha +{\left\|w\right\|}_{2}-\frac{1}{2\mu } \end{array}$$

at *t* = 1 − 1/(*μ*‖*w*‖_2_). The claim is proved.

(ii) Case 2. *μ*||*w*||_2_ ≤ 1

In this case, *h*′(*t*) = *μ*‖*w*‖_2_^2^(*t* − 1 + 1/(*μ*‖*w*‖_2_)) > 0 for all *t* > 0 and $\underset{t\to 0+}{\lim}h\left(t\right)=\left(\mu /2\right){\left\|w\right\|}_{2}^{2}+\alpha >\left(\mu /2\right){\left\|w\right\|}_{2}^{2}$. Thus, the minimizer is *z*_*opt*_ = 0.

Combining the results for *z* = 0 and *z* ≠ 0, we conclude that if (*μ*/2)‖*w*‖_2_^2^ ≤ *α* + ‖*w*‖_2_ − 1/(2*μ*) then, *z*_*opt*_ = 0; otherwise, *z*_*opt*_ = max{1 − 1/(*μ*‖*w*‖_2_), 0}*w*.

Next, we look for conditions under which the inequality (*μ*/2)‖*w*‖_2_^2^ ≤ *α* + ‖*w*‖_2_ − 1/(2*μ*) holds. Substituting *r* = ||*w*||_2_ into the inequality, we consider the equation

(21) $$\begin{array}{c} q\left(r\right)≔\frac{\mu }{2}r^{2}-r-\left(\alpha -\frac{1}{2\mu }\right)=0 \end{array}$$

in an unknown *r*. The zeros of the equation are given by $r_{1,2}=\left(1/\mu \right)\left(1\mp \sqrt{2\mu \alpha }\right)$. So *q*(*r*) ≤ 0 for *r* ∈ [*r*_1_, *r*_2_], where *r*_1_ ≤ 0 for 2*μα* > 1, and *q*(*r*) > 0 for *r* > *r*_2_. Therefore, for 2*μα* > 1, if ${\left\|w\right\|}_{2}\leq \left(1/\mu \right)\left(1+\sqrt{2\mu \alpha }\right)$, we have (*μ*/2)‖*w*‖_2_^2^ ≤ *α* + ‖*w*‖_2_ − 1/(2*μ*), and consequently, *z*_*opt*_ = 0; otherwise, (*μ*/2)‖*w*‖_2_^2^ > *α* + ‖*w*‖_2_ − 1/(2*μ*), and consequently, *z*_*opt*_ = max{1 − 1/(*μ*‖*w*‖_2_), 0}*w*.

With a threshold $\kappa =\left(1/\mu \right)\left(1+\sqrt{2\mu \alpha }\right)$, if ‖*w*‖_2_ ≤ *κ*, then *z*_*opt*_ = 0; otherwise, *z*_*opt*_ = max{1 − 1/(*μ*‖*w*‖_2_), 0}*w*. Using the HT operator in (14), we have *z*_*opt*_ = max{1 − 1/(*μ*‖*w*‖_2_), 0}*H*_*κ*_(*w*). We complete the proof of the theorem.

Remark. In practice, the condition 2*μα* > 1 for nonzero *α* in Theorem 1 is fulfilled. Now we summarize our algorithm as follows.

## 3. Results and Discussion

In this section, the performance of the proposed algorithm is compared with that of the *l*_1_-norm regularization algorithm. Both algorithms are implemented in MATLAB using the NADA and tested with the 2D Shepp-Logan phantom and a cardiac image [21] of size 128 × 128 on an Intel Core i7 3.40 GHz PC. In each test, the same system *u* = *Φf* + *e* is created, where *Φ* ∈ *R*^*m*×*n*^2^^ (*m* ≈ 0.3*n*^2^) is a random matrix and the noise *e* = 0.02∗mean(*Φf*)∗randn(*m*). The weight parameter *α* is chosen as 0 and 1 for the *l*_0_-norm regularization and the proposed algorithm, respectively. Other two important parameters in the objective function are taken as *β* = 2^8^, *μ* = 2^4^ for the Shepp-Logan phantom and *β* = 2^7^, *μ* = 2^2^ for the cardiac image, respectively.

**Figure 1:**
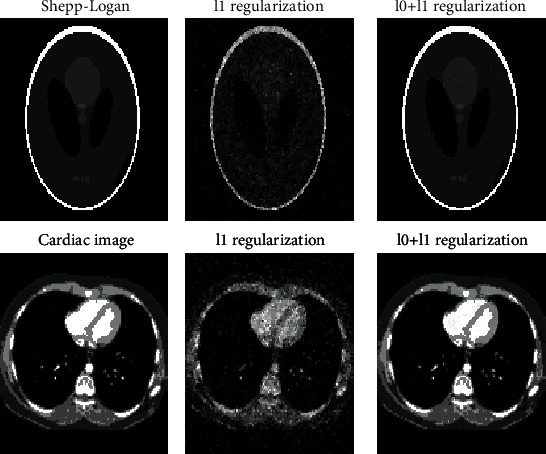
Original and reconstructed images. First row: Shepp-Logan phantom after 200 iterations. Second row: cardiac image after 500 iterations.

Experiments are conducted to compare the reconstruction by the two algorithms after the same number of iterations. The original and reconstructed images for two images after the same numbers of iterations are shown in Figure 1. The images demonstrate that the proposed algorithm yields much better reconstruction. The quality of reconstructed images is compared using several commonly used criteria: the relative error, the root-mean-square error (RMSE), the normalized root mean square deviation (NRMSD), the normalized mean absolute deviation (NMAD), and the structural similarity index (SSIM). The experimental results from 100 tests are summarized in Table 1. It shows that the proposed algorithm produces significantly improved evaluation measurement: 88%–167% for the Shepp-Logan phantom and 67%–114% for the cardiac image, respectively, while taking less CPU time. Overall, the new algorithm provides better accuracy after the same iteration number.

**Figure 2:**
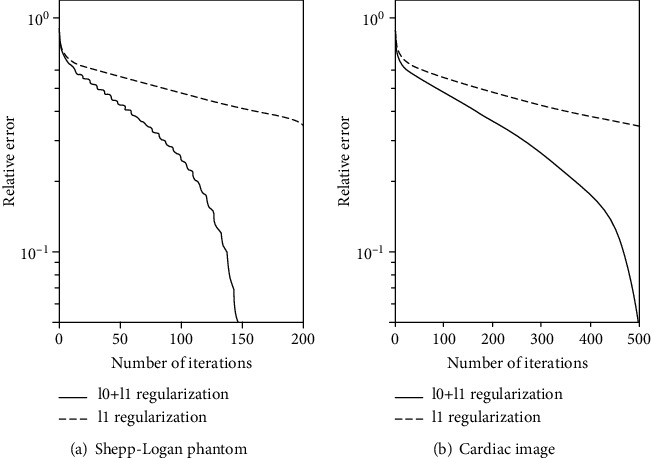
Relative error v.s. iteration number in reconstruction by two algorithms. (a) Shepp-Logan phantom. (b) Cardiac image.

Figure 2 shows the graph of relative error v.s. the number of iterations for the two algorithms. The curves indicate that the proposed algorithm requires much less iterations and time to achieve the same accuracy and yields much faster convergence. After a certain number of iteration numbers, the relative error of the proposed algorithm drops sharply, while the relative error of the *l*_1_-norm regularization is slowly decreasing and does not improve much over the iterations.

The proposed regularization model introduces an *l*_0_-norm term in addition to an *l*_1_-norm term. The *l*_0_-norm of a vector is the number of nonzero elements in the vector; thus, the *l*_0_-norm is appropriate for representing sparsity. Meanwhile, the *l*_0_-norm term makes the model less smoothing. Numerical experiments demonstrate that, in the proposed model, the edge is better preserved, and a better sparsity representation is provided. So, the proposed algorithm improves the quality and the efficiency of the reconstruction even with the extra computation from the added *l*_0_-norm term.

The effect of the weight parameter *α* of the *l*_0_-norm is tested. From our experiments, the parameter *α* from 0.5 to 1 almost does not not affect the efficiency of the algorithm. Selecting good values of other parameters *β*, *μ*, and *γ* in Minimization (10) is a challenging problem and will be further investigated in the future.

High-quality CT image reconstruction from limited projection data is challenging. Learning based algorithms such as structure-aware sparse Bayesian learning [22] could yield improved performance in reconstructing tomographic images from limited data, since structural prior knowledge is exploited. Enhancing the proposed algorithm in a learning-based framework using prior knowledge will be the topic of future research.

## Figures and Tables

**Algorithm 1:**
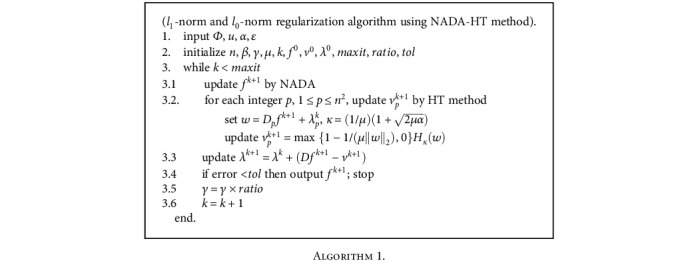
Algorithm 1.

**(a):** 

Shepp-Logan phantom, after 200 iterations						
	Time(s)	Error	RMSE	NRMSD	NMAD	SSIM
*l*_1_ regularization	53	0.341	0.083	0.086	0.422	0.356
*l*_0_ + *l*_1_ regularization	52	0.035	0.009	0.009	0.033	0.949

**(b):** 

Cardiac image, after 500 iterations						
	Time(s)	Error	RMSE	NRMSD	NMAD	SSIM
*l*_1_ regularization	88	0.362	0.111	0.076	0.352	0.442
*l*_0_ + *l*_1_ regularization	83	0.119	0.014	0.010	0.041	0.936

## Data Availability

The data used to support the findings of the study included within the article.
